# The Occurrence of Dropped Head Syndrome After a Cervical Medial Branch Nerve Block in a Patient With Cervicothoracic Kyphotic Deformity: A Case Report

**DOI:** 10.7759/cureus.61586

**Published:** 2024-06-03

**Authors:** Adewale Adeniran, Emily C Courtois, Donna D Ohnmeiss

**Affiliations:** 1 Orthopedic Surgery, Texas Back Institute, Fort Worth, USA; 2 Research, Texas Back Institute, Plano, USA; 3 Research Foundation, Texas Back Institute, Plano, USA

**Keywords:** deformity, kyphosis, cervical spine, medial branch nerve block, dropped head syndrome

## Abstract

Complications from medial branch blocks (MBBs) are rare when following proper procedural protocol. Dropped head syndrome (DHS) is characterized by profound muscle weakness in the cervical spine, resulting in a failure to maintain a level horizontal gaze and, in the worst cases, a chin-on-chest deformity. In this case report, we described DHS developing after cervical MBBs using short-acting anesthetic agents and subsequent management. A 69-year-old woman with a previous C6-C7 anterior cervical discectomy and fusion (ACDF) underwent bilateral posterior cervical MBBs targeting the C4-C5 and C5-C6 levels. Immediately following the injection, she reported a sudden inability to lift her head and was subsequently diagnosed with DHS. This condition continued with minimal improvement for over six months. After weighing the risks, the patient elected to avoid surgery, and she was provided a soft cervical collar and prescribed physical therapy. DHS is a debilitating condition more commonly associated with neurodegenerative conditions and inflammatory myopathy, which has received limited attention due to its rarity as a complication of cervical radiofrequency neurotomy. Surgery for this condition, when considered, typically involves long-segment posterior cervical instrumented fusion. Undergoing such a surgery is a complicated discussion that should consider patient clinical factors and preferences. The clinical impact of loss of strength in paraspinal musculature in this patient population is clearly deserving of further study.

## Introduction

Chronic neck pain is prevalent and is more common in females globally [1,2]. Cervical facet joint arthropathy is a common cause of this pain, but establishing this diagnosis is challenging due to non-specific symptoms and a lack of reliable diagnostic tools [3]. Radiofrequency ablation (RFA) of the medial branches can provide temporary pain relief in well-selected patients [4,5]. A positive response to multiple medial branch blocks (MBBs) is typically required prior to undergoing RFA [6,7]. Reported complications from MBBs are rare when following proper procedural protocol [8,9].

Dropped head syndrome (DHS) is characterized by profound muscle weakness in the cervical spine, resulting in a failure to maintain a level horizontal gaze and, in the worst cases, a chin-on-chest deformity. In several case reports, DHS has been described as a consequence of the denervation of the cervical paraspinous musculature after cervical RFA [9]. In this case report, we describe the development of DHS after undergoing cervical MBBs using short-acting anesthetic agents and the subsequent management of this condition [10,11]. The patient provided HIPAA-compliant permission to use her clinical information in this report.

## Case presentation

Initial presentation

**Figure 1:**
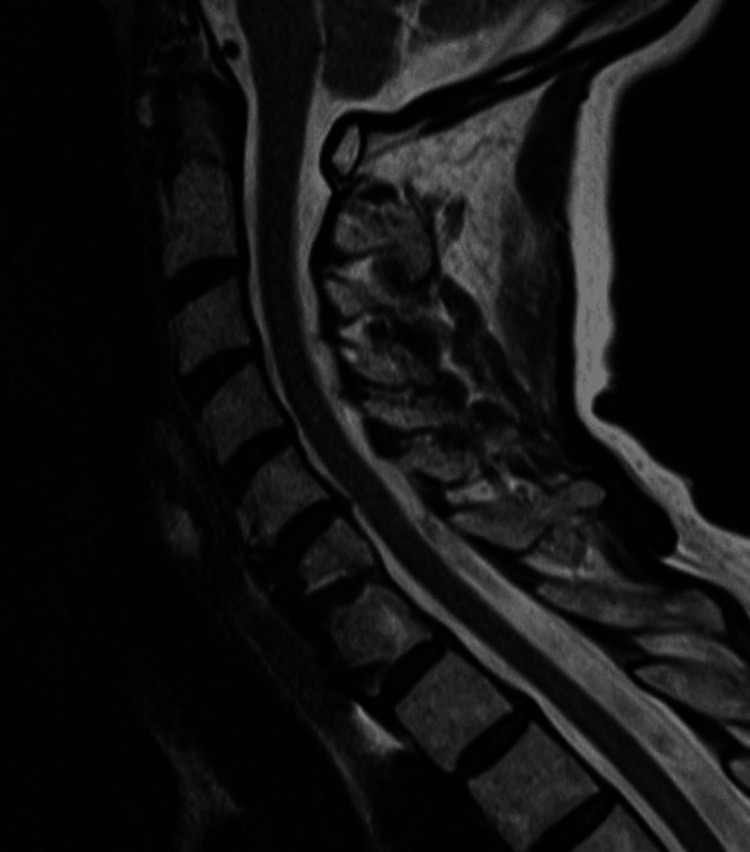
T2-weighted MRI of patient’s mid-sagittal cervical spine approximately two months before medial branch block injection.

A 69-year-old woman with previous C6-C7 anterior cervical discectomy and fusion (ACDF) and pre-existing cervicothoracic kyphotic deformity related to osteoporotic compression/wedge fractures at T6 and T7 (Figure 1) presented to her pain physician with persistent neck pain and was offered cervical MBBs as a precursor to undergoing cervical RFAs. She had a history of smoking, mitral valve prolapse, multiple transient ischemic attacks (TIAs), and previous strokes.

**Figure 2:**
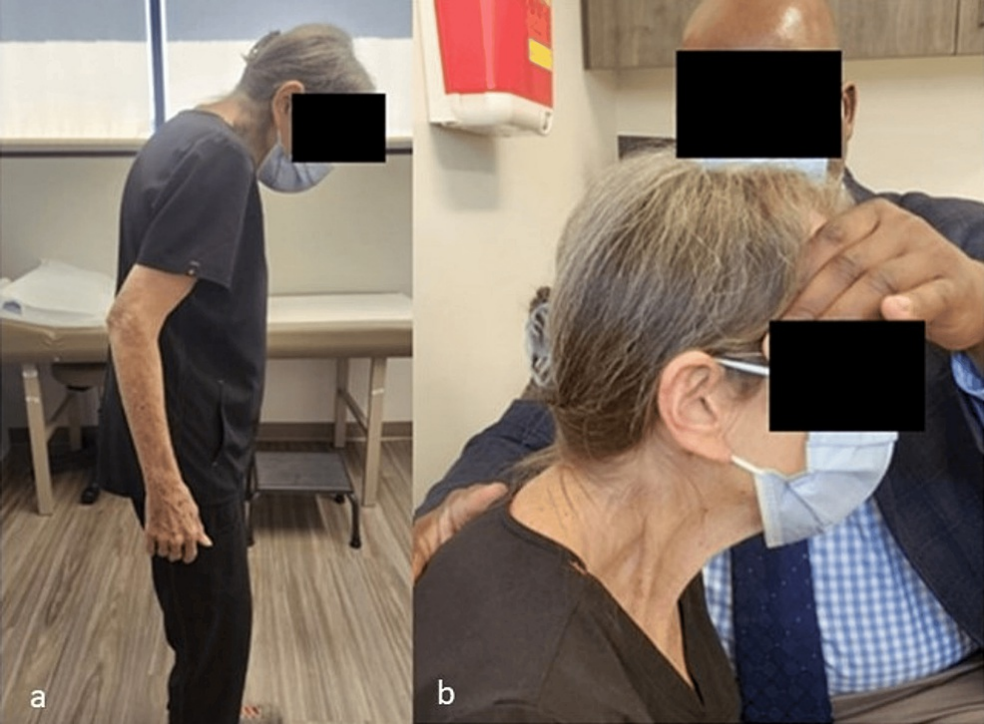
(a) Full-length sagittal photo of the patient upon physical examination; (b) photo of manual extension of the cervical spine could demonstrate the patient’s level gaze.

She then underwent bilateral posterior cervical MBBs targeting the C4-C5 and C5-C6 levels. The procedural report described no complications. She reported an almost immediate inability to lift up her head after the conclusion of the procedure. A month and a half following the injections, she presented to a spine surgeon, describing increased neck pain and perpetual but ineffectual straining to hold her head up. She was obliged to manually lift up her head to participate in normal activities, including feeding herself. A physical exam demonstrated obvious chin-to-chest posture with compensatory extension of the hips and flexion of the knees, which contributed to immense fatigue (Figure 2a). The neck could be manually extended to maintain a level gaze (Figure 2b).

The risks and benefits of the reconstructive surgery that would be necessary to treat her DHS were discussed, and the patient elected a trial of non-operative treatment with close follow-up. She was provided a soft cervical collar and prescribed physical therapy.

Imaging

**Figure 3:**
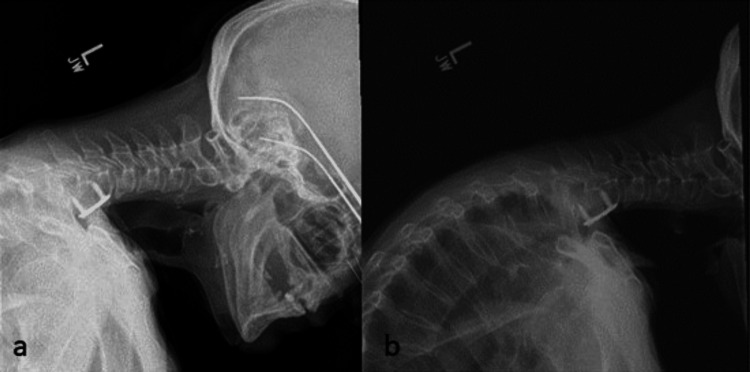
(a) X-ray of patient in relaxed lateral position for initial clinic X-rays six weeks post DHS onset; (b) X-ray of patient in extension for initial clinic visit six weeks post DHS onset.

**Figure 4:**
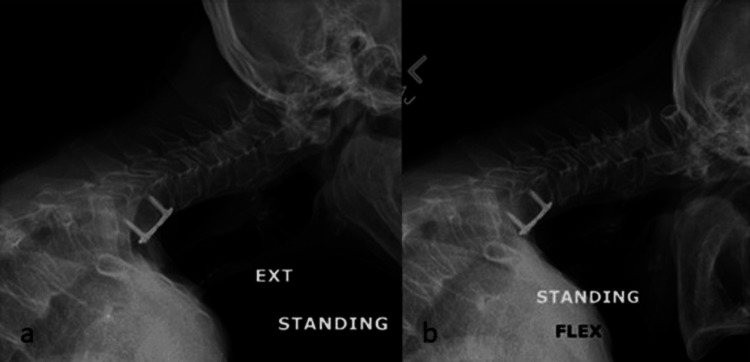
(a) X-ray of patient in extension four months post DHS onset; (b) X-ray of patient in flexion four months post DHS onset.

**Figure 5:**
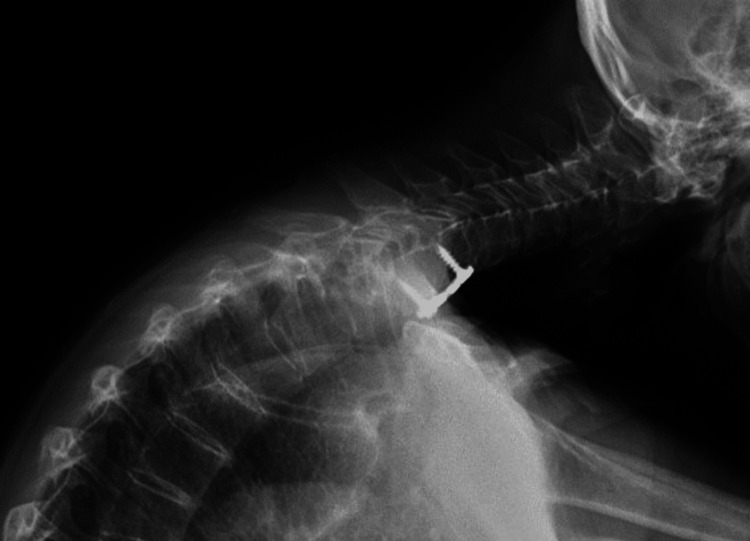
X-ray of patient in relaxed lateral position six months post DHS onset.

A progression of the patient’s imaging from six weeks through six months was collected and displayed in Figures 3a, 4, 5 over the course of several months after the onset of DHS.

Subsequent evaluation

An evaluation two months following the onset of DHS demonstrated no significant change in her condition. Two weeks later, an MRI of the brain without contrast obtained showed no evidence of acute intracranial abnormality despite nonspecific mild global intracranial atrophy, mild cerebral white matter, and pontine disease. An MRI of the cervical spine showed unchanged findings when compared to images from five months prior. No spinal cord compression was identified. There were no signal changes in the cervical paraspinal musculature.

EMG/nerve conduction studies were obtained approximately three months post-DHS onset and demonstrated active denervation isolated to bilateral cervical paraspinals without evidence of cervical radiculopathy or a neurodegenerative process. Upon reevaluation a week later, the patient reported mild improvement, reporting that she could hold her head up for a maximum of 15 minutes at a time. Her quality of life was most impacted by her inability to drive due to her inability to look at the mirrors bilaterally. Given her improvements, surgery was deferred.

At four months post-DHS onset, she was reevaluated, reporting continued improvements in her neck strength. She had continued physical therapy.

Approximately five months post-DHS onset, she was reevaluated and reported no significant improvements, with continued limitations in the performance of activities of daily living. She was encouraged to continue physical therapy.

At six and a half months post-DHS onset, she returned to the clinic, reporting improvement in her capacity to lift her head up. She had persistent neck pain, which was managed by pain medications. Full-length X-rays were reviewed and demonstrated hyperinflated, emphysematous lungs as well as global osteopenia. The images also demonstrated the C2-C7 sagittal vertebral axis at 62 mm and the C2-C7 Cobb angle at 22° kyphosis. Surgery was offered, and the patient expressed a strong preference to avoid it once surgical options and risks were shared. She elected to continue physical therapy and non-surgical pain treatments. At her most recent follow-up, 11 months after the onset of her condition, she expressed continued improvements in her ability to extend her neck and could tolerate being out of her soft collar for up to three hours at a time before fatigue caused her to use the collar. She continued home exercises and retained a desire to avoid surgery.

## Discussion

DHS is a debilitating condition more commonly associated with neurodegenerative conditions and inflammatory myopathy, which has previously been reported in three case reports as a rare complication of cervical radiofrequency neurotomy [9,12,13]. This case, as well as the case reports that describe DHS as a consequence of RFAs, highlight the anatomic fact that the dorsal ramus of our spinal nerves is a mixed nerve and provides both sensation and motor fibers to the paraspinous musculature, facet joints, and the skin overlying the back. A recent anatomical study confirmed that medial branches follow the same general pathway in the cervical spine, originating from the dorsal root of the spinal nerve, following the pedicles, and heading towards target muscles: the multifidus and semispinalis cervicalis muscles [14]. Extensive connective fibers were identified between multiple medial branches. In some cases, direct facet joint branches were identified as arising directly from the dorsal root of the spinal nerve and course into the anterior joint capsule [14]. Lesioning, whether through ablation or chemical treatment of the medial branches, necessarily results in weakness of the paraspinals to some extent [15]. The extent to which medial branch denervation and resulting paraspinal weakness are permanent and the clinical impact of such paraspinal denervation are unclear from the literature.

Muscle denervation is known to result in early and irreversible fatty degeneration, and medial branch lesioning necessarily results in muscle denervation. Nevertheless, there are very few small studies that clearly demonstrate clinically relevant weakness or muscle atrophy. There are sparse studies on the effect of RFAs on the progression of spinal instability conditions such as lumbar spondylolisthesis or cervical kyphosis. Further work is necessary in this area.

While denervation of these medial branches necessarily results in paraspinal muscle weakness in all patients to some extent, the clinical impact of this weakness likely depends on patient factors. The patient in our case was a frail, somewhat debilitated woman with multiple medical co-morbid conditions and pre-existing cervicothoracic kyphosis. Having lost the upright alignment of her upper spine, her cervical paraspinal musculature played a critical role in the maintenance of horizontal gaze. Losing strength in these critical structures resulted in a significant, long-lasting decrement in her quality of life, with little certainty as to her ultimate outcome. The clinical impact of loss of strength in paraspinal musculature in this patient population is clearly deserving of further study.

## Conclusions

In this case report, we describe a patient who suffered DHS as a consequence of cervical MBBs. To the best of our knowledge, such a complication has never been reported. DHS is frequently transient, and treatment is typically supportive, including physical therapy and bracing. Surgery, when considered, typically involves long-segment posterior cervical instrumented fusion. Undergoing such a surgery is a complicated discussion that should consider patient clinical factors and preferences. In this case, surgery was considered for our patient, who persists in experiencing life-altering weakness of the posterior cervical musculature more than eight months after her MBBs. Her comorbid conditions of cervical thoracic scoliosis, smoking history, mitral valve prolapse, multiple TIAs, and previous stroke all increase the risk of nonunion, hardware failure, and perioperative demise, and we have mutually decided to forgo surgical treatment of her condition. Nevertheless, she has suffered significant decrement in her quality of life, pain, and mobility. Based on this clinical experience, we encourage caution when considering either MBBs or RFAs in patients with cervicothoracic kyphosis.
